# Anti-Inflammatory, Antioxidant, and Antifibrotic Effects of Kefir Peptides on Salt-Induced Renal Vascular Damage and Dysfunction in Aged Stroke-Prone Spontaneously Hypertensive Rats

**DOI:** 10.3390/antiox9090790

**Published:** 2020-08-26

**Authors:** Yu-Hsuan Chen, Hsiao-Ling Chen, Hueng-Chuen Fan, Yu-Tang Tung, Chia-Wen Kuo, Min-Yu Tu, Chuan-Mu Chen

**Affiliations:** 1 Department of Life Sciences, and Ph.D. Program in Translational Medicine, National Chung Hsing University, Taichung 402, Taiwan; yhchen1218@smail.nchu.edu.tw (Y.-H.C.); fanhuengchuen@yahoo.com.tw (H.-C.F.); f91625059@tmu.edu.tw (Y.-T.T.); kuochiawen@yahoo.com.tw (C.-W.K.); du0807@yahoo.com.tw (M.-Y.T.); 2 Ph.D. Program in Tissue Engineering and Regenerative Medicine, National Chung Hsing University, Taichung 402, Taiwan; 3 Ph.D. Program in Tissue Engineering and Regenerative Medicine, National Health Research Institutes, Taichung 402, Taiwan; 4 Department of Biomedical Sciences, Da-Yeh University, Changhwa 515, Taiwan; bellchen@mail.dyu.edu.tw; 5 Department of Pediatrics, Tungs’ Taichung Metroharbor Hospital, Wuchi, Taichung 435, Taiwan; 6 Department of Medical Research, Tungs’ Taichung Metroharbor Hospital, Wuchi, Taichung 435, Taiwan; 7 Department of Rehabilitation, Jen-Teh Junior College of Medicine, Miaoli 356, Taiwan; 8 Institute of Metabolism and Obesity Sciences, Taipei Medical University, Taipei 110, Taiwan; 9 Department of Internal Medicine, Taichung Armed Forces General Hospital, Taichung 411, Taiwan; 10 Department of Orthopaedic Surgery, Taichung Armed Forces General Hospital, Taichung 411, Taiwan; 11 Aviation Physiology Research Laboratory, Kaohsiung Armed Forces General Hospital Gangshan Branch, Kaohsiung 820, Taiwan; 12 The iEGG and Animal Biotechnology Center, and Rong Hsing Research Center for Translational Medicine, National Chung Hsing University, Taichung 402, Taiwan

**Keywords:** kefir peptides, chronic kidney disease, glomerulosclerosis, stroke-prone spontaneously hypertensive rats, renal dysfunction

## Abstract

The increased prevalence of renal dysfunction and chronic kidney disease (CKD) and the high costs and poor outcomes of treatment are a significant health issue. The consequence of chronic high blood pressure is the increased prevalence of target organ end-stage renal disease, which has been proven to be a strong independent risk factor for adverse cardiovascular disease. A previous study showed that kefir products have anti-inflammatory and anti-hypertensive activities and immunological modulation functions. However, no data regarding the beneficial effects of kefir peptides (KPs) on salt-induced renal damage or related kidney diseases are available. In this study, KPs were orally administered to aged salt-induced stroke-prone spontaneously hypertensive (SHRSP) rats, and the effects of KPs against inflammation and oxidative stress and their ability to protect against renal dysfunction were evaluated. Fifty-five-week-old SHRSP rats under induction with 1% NaCl in drinking water for 4 weeks showed multiple renal injuries with increased renal inflammation, fibrosis, oxidative stress, tubular atrophy, and glomerulosclerosis. In contrast, oral gavage with KPs reduced the urine protein to creatinine (UPC) ratio, the fractional excretion of electrolytes (FeNa and FeCl), extracellular matrix deposition, and the interstitial fibrotic α-smooth muscle actin (α-SMA) levels in salt-induced SHRSP rats. The renal infiltration of inflammatory cells; the release of monocyte chemoattractant protein-1 (MCP-1), vascular cell adhesion molecule-1 (VCAM-1), endothelin-1 (ET-1), and the cytokine nucleotide-binding oligomerization domain (NOD)-like receptor family pyrin domain containing 3 (NLRP3) and transforming growth factor-β (TGF-β); the reactive oxygen species (ROS) levels; and histopathological lesions were also decreased in salt-induced SHRSP rats. Furthermore, KP treatment significantly increased the renal superoxide dismutase (SOD) activity and the glomerular filtration rate (GFR), which exerted potent protection against salt-induced chronic kidney disease in SHRSP rats. The results of this study suggest that KPs ameliorate salt-induced renal damage, tubular atrophy, and glomerular dysfunction through anti-inflammatory, antioxidative stress, and antifibrotic activities, and might be a promising protective agent against high salt-induced renovascular-related diseases.

## 1. Introduction

Chronic kidney disease (CKD) is a worldwide public health threat. A high salt intake may increase the urine protein levels and is an important risk factor for reduced kidney function [1]. Moreover, high salt intake was demonstrated to exacerbate kidney disease in people who already show kidney problems [2]. In CKD patients, the risk of blockade of the blood supply to the brain or heart, resulting in stroke or cardiovascular disease (CVD), is significant. Increasing evidence shows that CKD is a key risk factor for CVD and stroke, independent of traditional risk factors, such as hypertension, hyperlipidemia and diabetes [3]. Gelosa et al. [4] also reported a higher risk of stroke in CKD, even after traditional risk factors were improved, which may be because CKD and cerebrovascular disease arise from a common vascular origin. In dialysis patients, the mortality rates due to CVD are 10 to 30 times higher than those in the general population [5]. In high-risk patients with CVD, less severe kidney disease is an independent risk factor for CVD outcome [6,7].

In vivo, in isolated perfused kidneys and in kidneys perfused in situ, hyperchloremia was found to result in severe renal vasoconstriction and a marked decrease in the glomerular filtration rate (GFR) [8]. Schmidlin et al. [9] also suggested that tubuloglomerular feedback (TGF) is activated by increased chloride levels, leading to increased renal afferent arteriolar resistance, decreasing renal blood flow and GFR, and increasing systemic arterial pressure [2]. In spontaneously hypertensive (SHR) rats, salt-induced hypertension was found to be associated with blunted renal sympathoinhibition [10,11] and reduced natriuresis and diuresis [12]. Spontaneously hypertensive stroke-prone (SHRSP) rats are a unique animal model and show inflammation, hypertension, proteinuria, and histological lesions in the renal vasculature and parenchyma [4]. Grisk et al. [1] demonstrated that SHRSP rats given a high-salt diet developed severe hypertension and nephrosclerosis. In the present study, we generated salt-induced CKD in aged SHRSP rats by the administration of 1% NaCl in the drinking water to develop an animal model of hypertension-related renovascular damage and kidney disease.

Kefir, a unique cultured dairy product in which combined lactic acid and lactose fermentation takes place in milk produced by kefir grains, has been used clinically to treat gastrointestinal disease, hypertension, ischemic heart disease, and allergies [13,14]. Several studies have demonstrated the numerous biological activities of kefir peptides (KPs), including their antibacterial, antioxidant, anti-diabetic, anti-thrombotic, anti-osteoporotic, and immunostimulating effects [15,16,17]. Baum et al. [18] reported eight peptides derived from kefir fermented milk protein with an inhibitory effect on angiotensin-converting enzyme (ACE). Currently, ACE inhibitors are the standard of treatment for CKD patients. Therefore, KPs may improve high salt-induced renovascular-related kidney diseases. However, the use of KPs to prevent CKD has not been well studied. In this study, the protective effects of kefir peptides against CKD in NaCl-induced kidney disease were intensely investigated in SHRSP rats for the first time.

## 2. Materials and Methods

### 2.1. Kefir Peptides

The KPs used in this study were purchased from Phermpep Co., Ltd. (Taichung, Taiwan). The kefir-fermented product derived from a milk protein source used in this study to determine the potential role of KPs in the management of salt-induced chronic kidney disease contained 15.48 g/100 g peptide, and its main components are listed in Supplementary Table S1. The reproducibility of the prepared kefir peptides was high, as shown in our previous study [16,19,20].

### 2.2. Animal Experiment

Male Wistar-Kyoto (WKY/NCrlNarl) rats (aged 50–52 weeks) were purchased from the National Laboratory Animal Center (Taipei, Taiwan). Spontaneously hypertensive stroke-prone (SHRSP/Ngsk) rats were obtained from Kyoto University (Kyoto, Japan) [21] and bred through random mating at the Animal Center of National Chung Hsing University. The rats were kept in an SPF-grade animal facility under a 12 h light/dark cycle at 24 ± 2 °C under 50–60% humidity and given access to regular rodent chow (Altromin 1324; Altromin GmbH, Lage, Germany) and water ad libitum [22,23]. This study was conducted according to institutional guidelines and approved by the Institutional Animal Care and Utilization Committee (IACUC Approval No. 103-78) of National Chung Hsing University, Taiwan.

Male WKY rats were assigned to a Normal group (*n* = 5). Other male SHRSP rats (aged 50–52 weeks) were randomly assigned into three treatment groups: the (1) SHRSP group (*n* = 5), (2) the SHRSP+NaCl group (*n* = 6), and (3) the SHRSP+NaCl+KPs group (*n* = 6). Rats in the SHRSP+NaCl and SHRSP+NaCl+KPs groups were administered 1% NaCl in their drinking water, while rats in the SHRSP group were not given 1% NaCl. Rats in the SHRSP+NaCl+KPs group were orally administered kefir peptides diluted in ddH_2_O (200 mg/kg body weight/day) for 4 weeks during the same period of 1% NaCl drinking water supplement; the KP dose used in this study was determined by a dose-dependent KPs pretreatment result, as shown in Supplementary Figure S1. Meanwhile, rats in the other two groups (Normal and SHRSP) were given the same volume of ddH_2_O. Subsequently, each rat was anesthetized, and the kidneys were immediately removed, rinsed in ice-cold 0.90% (*w*/*v*) NaCl, blotted dry, and weighed. All the samples were stored at −80 °C for further assays.

### 2.3. Determination of Serum and Urine Biochemical Markers

Blood urea nitrogen (BUN) and creatinine in the blood and FeNa, FeK, and FeCl in the urine were measured using the ADVIA 1800 Chemistry System (Siemens Healthcare, Erlangen, Germany), as described previously [24]. For urinalysis, the rats were placed in metabolic cages with free access to tap water, and urine was collected overnight. The urine protein, creatinine, and the urine protein-to-creatinine ratio (UPC) were quantified using an IDEXX Vet Test Analyzer (IDEXX Lab, Inc., Westbrook, ME, USA).

### 2.4. Measurement of the Glomerular Filtration Rate (GFR) of Plasma FITC-Inulin

GFR measurements were performed in conscious rats by determining the plasma clearance of FITC-labeled inulin (50 mg/mL in 0.85% NaCl, 2 μL/g body weight) (TdB Consultancy AB, Uppsala, Sweden) following single-dose intravenous injection via the tail vein, as described previously [25]. Briefly, the rats were anaesthetized with Zoletil and Rompun, and 600 μL of blood was collected at 120 min after a single injection of fluorescein isothiocyanate (FITC)-labeled inulin. The plasma was separated, and the inulin clearance was quantified by the FITC intensity. Fluorescence measurements were performed using the PARADIGM Detection Platform (Beckman Coulter, Inc., Brea, CA, USA). The GFR was calculated using a two-compartment model of two-phase exponential decay (GraphPad Prism 6, San Diego, CA, USA).

### 2.5. Histopathological Examination of the Kidneys

After sacrificing the rats, the upper lateral half of the kidney tissue was freshly dissected. These tissues were fixed in 10% buffered formaldehyde (pH 7.0), embedded in paraffin, sectioned into 3 μm sections, and processed for three types of histopathological stains: hematoxylin and eosin (H&E) [19], Masson’s trichrome [20] and Sirius red [25]. The severity of the observed lesions was graded by a pathologist according to the standard method [26]. In addition, 3 to 6 kidney arteries from each animal were evaluated. The relative renal interlobular artery area (%) and renal interlobular lumen (µm^2^) were calculated according to previous reports [27,28].

### 2.6. Immunohistochemical Staining

Formaldehyde-fixed and paraffin-embedded sections were cut at a thickness of 5 μm. The sections were incubated in 3% H_2_O_2_ for 10 min to block the endogenous peroxidase activity and then incubated overnight at 4 °C with primary antibodies against MCP-1 (ab7202, 1:100 dilution; Abcam, Cambridge, UK), α-SMA (A2547, 1:200 dilution; Sigma, St. Louis, MO, USA), and ET-1 (ab2786, 1:100 dilution; Abcam, Cambridge, UK). The negative control was incubated with normal serum instead of primary antibodies. All the sections were immunostained with the VECTASTAIN ABC kit (Vector Laboratories, Inc., Burlingame, CA, USA) in accordance with the manufacturer’s specifications.

### 2.7. Oxidative Stress and Inflammatory Activity Assays

The kidney samples were lysed with radioimmunoprecipitation assay (RIPA) buffer (EMD Millipore Corp., Billerica MA, USA) in PBS supplemented with protease inhibitor cocktail tablets (Roche, Inc., Indianapolis, IN, USA) at 200 mg/mL. The lysed samples were homogenized or sonicated on ice to remove insoluble particles and spun at 15,000× *g* for 30 min at 4 °C, and the supernatants were transferred to new tubes. The supernatants were subjected to a superoxide dismutase (SOD) activity measurement (Dojindo Molecular Technologies, Gaithersburg, MD, USA), a ROS/RNS assay (OxiSelect kit, STA-347, Cell Biolabs, San Diego, CA, USA) and inflammatory activity assay (BD^TM^ Cytometric Bead Array, cat. No. 558267, BD Biosciences, San Jose, CA, USA).

### 2.8. Western Blot Analysis

The total proteins from the tissue lysate (20 μg) were separated by 12% SDS-PAGE and electrotransferred onto polyvinylidene difluoride membranes. The membranes were blocked in 5% bovine serum albumin and then incubated with primary antibodies specific for ET-1 (ab2786, 1:2000 dilution; Abcam), α-SMA (A2547, 1:2000 dilution; Sigma), NF-κB (#8242, 1:3000 dilution; Cell Signaling Technology, Inc., Danvers, MA, USA), VCAM1 (ab134047, 1:1000 dilution; Abcam), TGF-β (ab92486, 1:3000 dilution; Abcam), Vinculin (ab129002, 1:5000 dilution; Abcam) and β-actin (ab119716, 1:5000 dilution; Abcam) overnight. After washing, the membranes were incubated with goat anti-rabbit IgG peroxidase-conjugated secondary antibody for 1 h. The membranes were developed using an enhanced chemiluminescence (ECL) Western blot detection system (GE Healthcare, Munich, Germany). The intensity of the bands was quantified by the ImageJ software.

### 2.9. Statistical Analysis

All the results are expressed as the mean ± SEM. Statistical analysis was performed using one-way ANOVA and Duncan’s post hoc test. *p* < 0.05 was used to indicate statistical significance.

## 3. Results

### 3.1. Effects of KPs on the Body Weight, Organ Index, and Biochemical Profiles of Salt-Treated SHRSP Rats

The body weight, kidney index (%), and biochemical data from the blood and urine of rats in the four different groups are shown in Table 1 and also in Supplementary Table S2. SHRSP rats fed 1% NaCl in water (SHRSP+NaCl) exhibited a lower body weight (202 ± 7.19 g) and higher kidney index (0.936 ± 0.076%), blood urea nitrogen (BUN; 31.7 ± 3.1 mg/dl), urine FeNa (1.47 ± 0.52%), FeCl (2.33 ± 0.67%), urine protein (UPRO; 901 ± 66.71 mg/dl), and urine protein-to-creatinine ratio (UPC; 49.3 ± 8.72 mg/dl) than those in the normal (WKY) rats (*p* < 0.05) and untreated SHRSP rats, indicating the progression of chronic kidney disease (CKD). However, the body weight, urine fractional excretion of electrolytes (FeNa and FeCl), UPRO, urine creatinine (UCRE), and UPC of salt-treated SHRSP rats were significantly improved by treatment with 200 mg/kg KPs for 4 weeks (SHRSP+NaCl+KPs group) compared to those of the SHRSP+NaCl rats (*p* < 0.05).

Furthermore, in this study to evaluate the renal glomerular filtration function, FITC-tagged inulin was injected through the tail vein, and the GFR clearance rate was measured. The results showed that salt-induced CKD rats (SHRSP+NaCl) had a significantly decreased glomerular filtration rate (GFR; 0.71 ± 0.49 mL/min/g) compared to that of normal (WKY) and untreated SHRSP rats (*p* < 0.05). However, the renal glomerular filtration function was restored in the salt-induced SHRSP (SHRSP+NaCl+KPs) group after KP treatment for 4 weeks (1.60 ± 0.13 mL/min/g; *p* < 0.05) (Table 1).

### 3.2. KPs Attenuated Salt-Induced Glomerulosclerosis and Tubular Damage in SHRSP Rats

**Figure 1:**
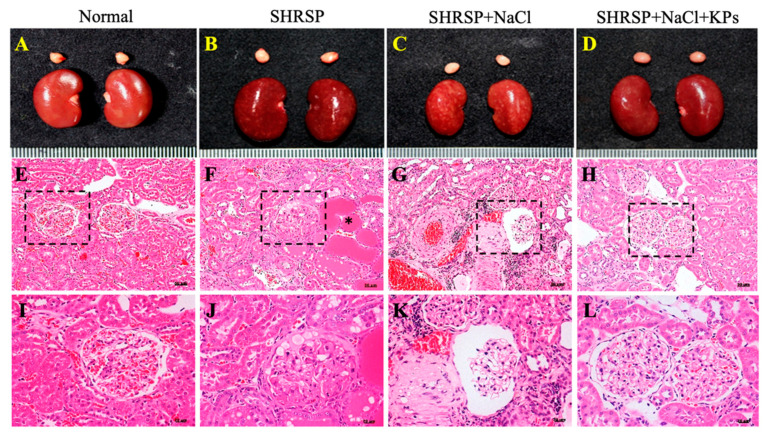
Exterior views of the kidneys of Wistar-Kyoto (WKY) and salt-induced SHRSP rats with or without kefir peptides (KPs) treatment and histopathological findings. The kidney exterior in normal WKY rats was smooth, plump, and reddish-brown (**A**). Kidneys with congestion, focal and firm lesions, and a coarse, irregular surface were observed in the untreated SHRSP rats (**B**), and the damage was even more severe in the kidneys of salt-treated SHRSP rats (**C**), but fewer or no gross lesions were observed in the salt-treated SHRSP rats administered KPs (**D**). As shown by hematoxylin and eosin (H&E) staining followed histopathological examination, (**E**) and (**I**) show normal kidney structures under 200× and 400× magnification, respectively. (**F**) and (**J**) show heterogeneous components of the glomeruli with some degenerative vacuole changes, hyaline cast formation (*), and moderate arteriolar hypertrophy in the juxtamedullary cortex with chronic progressive nephrosis under 200× and 400× magnification, respectively. (**G**) and (**K**) show tubular atrophy, tubular dilation, casts, hyperplasia, interstitial fibrosis, glomerulosclerosis, glomerular atrophy, interstitial inflammatory infiltrates, the Bowman’s space, and severe arterial wall thickening in the juxtamedullary cortex with chronic progressive nephrosis under 200× and 400× magnification, respectively. (**H**) and (**L**) show mild symptoms of chronic nephrosis. (**E**–**L**) Three fields were imaged for each individual.

The kidneys of normal rat were smooth, plump and reddish-brown (Figure 1A). The kidneys of rats in both the SHRSP (Figure 1B) and SHRSP+NaCl (Figure 1C) groups showed an irregular surface and a change in coloration. However, fewer or no gross lesions were observed in the SHRSP+NaCl+KPs group (Figure 1D). The histopathological results shown in Figure 1G,K indicate that salt-induced CKD caused tubular atrophy, interstitial fibrosis, glomerulosclerosis, glomerular atrophy, casts, and interstitial inflammatory infiltrates. Glomerular changes included the dilatation of the Bowman’s space (crescentic) with the atrophy of the capillary tuft. The levels of inflammatory cell infiltration and ischemic lesions were significantly increased after salt treatment, indicating advanced chronic progressive nephrosis. The oral administration of KPs reduced the levels of inflammatory cell infiltration and ischemic lesions due to kidney injury (Figure 1H,L). In addition, qualitative histopathological data were obtained by a pathology scoring system and are shown in Table 2. The data showed that the scores for afferent arteriolopathy, segmental glomerulosclerosis, and tubular atrophy in the juxtamedullary cortex were significantly higher in the SHRSP+NaCl group (2.3 ± 0.21) than both the SHRSP (1.3 ± 0.63) and SHRSP+NaCl+KPs (0.8 ± 0.17) groups (*p* < 0.05). In addition, the average scores for chronic progressive nephrosis, hypertrophy, and media in the arterioles were also significantly lower in the rats in the SHRSP+NaCl+KPs group than in those in the SHRSP+NaCl group (*p* < 0.05).

### 3.3. KPs Ameliorated Salt-Induced Interlobular Arterial Thickness in SHRSP Rats

**Figure 2:**
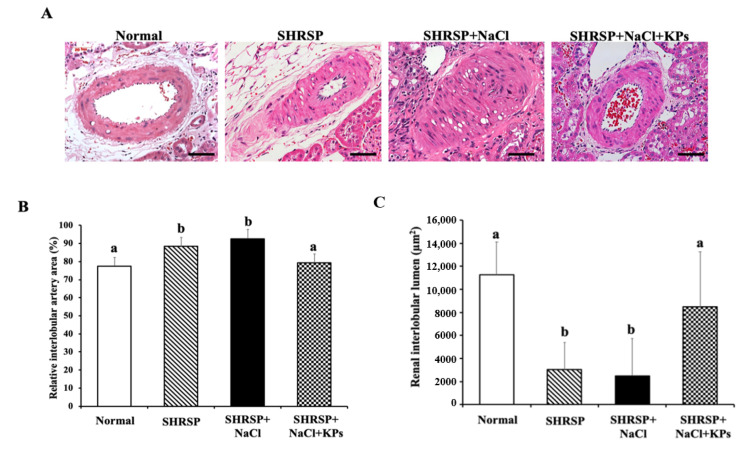
Morphological changes of the interlobular arteries in kidney tissues and quantitative total arterial thickness and lumen area. (**A**) WKY rats used as a normal control showed a normal morphology with a normal interlobular arterial thickness and lumen area. Interlobular arterial thickness was significantly elevated in the groups of SHRSP rats without (SHRSP) and with (SHRSP+NaCl) salt treatment compared to the WKY rats. The interlobular arterial thickness and lumen area in the SHRSP+NaCl+KPs group were examined by H&E staining at 400× magnification; scale bar = 50 µm. Three fields were imaged for each individual. (**B**) Interlobular arterial thickness (%) and (**C**) renal interlobular arterial lumen area (µm^2^) in the kidney tissues were quantified with ImageJ analysis software. Data are presented as the means ± SEMs (Normal, *n* = 5; SHRSP, *n* = 5; SHRSP+NaCl, *n* = 6; SHRSP+NaCl+KPs, *n* = 6) and were analyzed by one-way ANOVA. Data within columns that are not labeled with the same letter are significantly different (*p* < 0.05), as determined using Duncan’s test.

Histopathologic slides and the total H&E-stained areas of the interlobular arteries in the kidney tissues of rats in the Normal (WKY), SHRSP, SHRSP+NaCl, and SHRSP+NaCl+KPs groups are shown in Figure 2A. The quantitative total interlobular arterial wall thickness was significantly elevated to 90.9% ± 1.6% in the salt-induced SHRSP rats compared with the WKY rats (75.1% ± 1.9%; *p* < 0.05). However, the interlobular arteries of rats in the SHRSP+NaCl+KPs group showed a significantly decreased interlobular arterial walls thickness of 78.2% ± 2.4% compared with that of salt-induced SHRSP rats (*p* < 0.05; Figure 2B). In contrast, the total renal interlobular arterial lumen area was significantly decreased in both the untreated and salt-treated SHRSP rats compared with the WKY rats (*p* < 0.05), but the interlobular arterial lumen area in the salt-treated SHRSP rats was maintained after the KPs administration (Figure 2C).

### 3.4. KPs Improved Salt-Induced Renal Interstitial Collagen Deposition in SHRSP Rats

**Figure 3:**
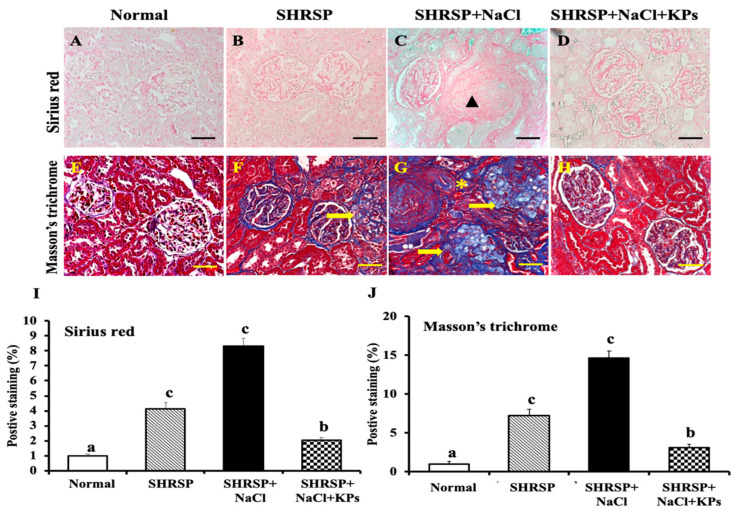
Sirius red and Masson’s trichrome staining of the kidney tissues of WKY and salt-induced SHRSP rats with or without KPs treatment. (**A**,**E**) No significant lesions were observed in the kidneys of WKY (normal) rats. The kidneys of rats in both the SHRSP (**B**,**F**) and SHRSP+NaCl (**C**,**G**) groups showed focal tubular degeneration, atrophy (*), glomerular fibrosis (yellow arrow), and arteriolar hypertrophy (black triangle). The cell structure and degree of interstitial fibrosis were analyzed at the glomerular sites (yellow arrow). (**D**,**H**) Less renal fibrosis was observed in the SHRSP+NaCl+KPs group. The results of the Sirius red staining show glial fibrillary structures (red; background color, green). The results of the Masson’s trichrome staining show glial fibrillary structures (blue) and muscle, cytoplasmic, and horny structures (red). Images were observed under 400× magnification. Scale bar = 50 μm. Three fields were imaged for each individual. (**I**) Quantitative analysis of the Sirius red-positive staining (%) and (**J**) Masson’s trichrome-positive areas (%) after staining was performed with ImageJ software. Data are presented as the means ± SEMs (Normal, *n* = 5; SHRSP, *n* = 5; SHRSP+NaCl, *n* = 6; SHRSP+NaCl+KPs, *n* = 6) and were analyzed by one-way ANOVA. Data within columns that are not labeled with the same letter are significantly different (*p* < 0.05), as determined using Duncan’s test.

The renal interstitial collagen deposition and fibrosis were assessed using Sirius red staining (Figure 3A–D) and Masson’s trichrome staining (Figure 3E–H), respectively. Masson’s trichrome staining of the kidney revealed salt-induced glomerulosclerosis, as well as tubulointerstitial, perivascular, and glomerular fibrosis in the SHRSP rats (Figure 3G), but the KP treatment for 4 weeks significantly reduced the extracellular matrix (ECM) accumulation in the SHRSP+NaCl+KPs group (Figure 3H). According to the results of the quantitative analyses of kidney sections stained with Sirius red (Figure 3I) and Masson’s trichrome (Figure 3J), both renal interstitial collagen deposition and fibrosis were significantly increased in the salt-treated SHRSP rats compared to rats in the SHRSP group (*p* < 0.05). However, the collagen deposition and fibrosis accumulation in the KPs-treated group were significantly improved.

### 3.5. KPs Decreased Salt-Induced Renal MCP-1, α-SMA, and ET-1 Expression in SHRSP Rats

**Figure 4:**
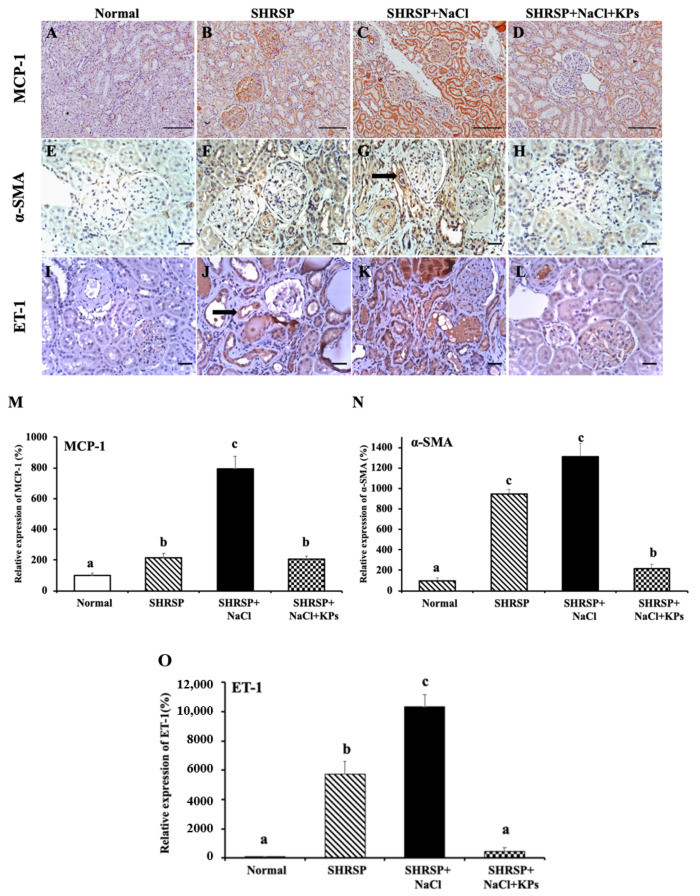
Effects of KPs on MCP-1, α-SMA, and ET-1 expression in the tubular cells and glomeruli of the kidneys of WKY and SHRSP rats, as determined by immunohistochemical (IHC) staining. The SHRSP+NaCl group (**C**) showed significantly more MCP-1 accumulation in tubular cells compared to WKY (normal) rats (**A**) and rats in the SHRSP group (**B**). The high MCP-1 expression in the SHRSP+NaCl group was reversed by the KPs treatment (**D**). Weak background staining for α-SMA was observed in the WKY (normal) rats (**E**). Positive IHC staining for α-SMA was noted in the smooth muscle of the venous neointimal tissue (black arrow) in the SHRSP+NaCl group (**G**), and more positive staining was observed in the SHRSP+NaCl group than in the SHRSP group (**F**). However, fewer α-SMA-positive renal cells were observed in the SHRSP+NaCl+KPs (**H**) group. Expressed E T-1 was concentrated in the cytoplasm of tubular epithelial cells (black arrow) in the SHRSP (**J**) and SHRSP+NaCl (**K**) groups. ET-1 expression was lower in the SHRSP+NaCl+KPs (**L**) group. (**A**–**D**) Images were observed under 200× magnification; scale bar = 50 µm. (**E**–**L**) Images were observed under 400× magnification; scale bar = 20 µm. Three fields were imaged for each individual. Quantitative analyses of the results of IHC staining for MCP-1 (**M**), α-SMA (**N**) and ET-1 (**O**) were carried out with ImageJ software. Data are presented as the means ± SEMs (Normal, *n* = 5; SHRSP, *n* = 5; SHRSP+NaCl, *n* = 6; SHRSP+NaCl+KPs, *n* = 6) and were analyzed by one-way ANOVA. Data within columns that are not labeled with the same letter are significantly different (*p* < 0.05), as determined using Duncan’s test.

Monocyte chemoattractant protein-1 (MCP-1) was examined as a chemotactic factor for macrophages. The positive MCP-1 immunohistochemical (IHC) staining for monocyte infiltration in renal tubular cells was significantly increased in the SHRSP+NaCl group compared with other groups (Figure 4A–D), and treatment with KPs markedly decreased the MCP-1 expression compared to that in the SHRSP+NaCl group (*p* < 0.05; Figure 4M). α-smooth muscle actin (α-SMA) is very useful for the detection of myofibroblasts in tissue (Figure 4E–H). The IHC staining for α-SMA showed the increased accumulation of myofibroblasts in both the glomeruli and the interstitium in the SHRSP+NaCl group (Figure 4G), but a significant reduction in the SHRSP+NaCl+KPs group (Figure 4H,N). Endothelin-1 (ET-1) is the most powerful endogenous vasoconstrictive peptide involved in the pathogenesis of CKD and CVD (Figure 4I–L). The ET-1 expression was significantly increased in the kidneys of the salt-treated SHRSP rats (Figure 4K) compared to those of the SHRSP rats fed normal water (*p* < 0.05), whereas the KPs administration to salt-treated SHRSP rats significantly decreased the total ET-1 expression (Figure 4L,O).

### 3.6. Effect of KPs on Oxidative Stress and Antioxidative SOD Activity in the Kidneys of Salt-Treated SHRSP Rats

**Figure 5:**
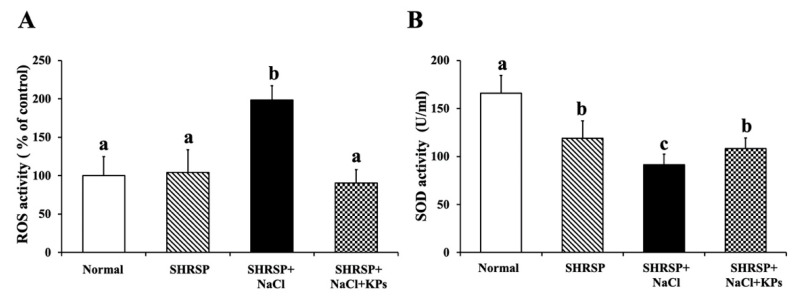
KPs treatment reduced the oxidative stress and increased the SOD activity in the kidneys of salt-sensitive SHRSP rats. (**A**) SHRSP rats in the SHRSP+NaCl group showed significantly increased kidney ROS accumulation. However, the treatment of salt-induced SHRSP rats with KPs reduced the ROS content to the level of that observed in the control WKY rats. (**B**) SHRSP rats in the SHRSP+NaCl group showed a significantly reduced SOD activity, but this activity was restored in the SHRSP+NaCl+KPs group due to the KPs administration. Data are presented as the means ± SEMs (Normal, *n* = 5; SHRSP, *n* = 5; SHRSP+NaCl, *n* = 6; SHRSP+NaCl+KPs, *n* = 6) and were analyzed by one-way ANOVA. Data within columns that are not labeled with the same letter are significantly different (*p* < 0.05), as determined using Duncan’s test.

In the normal group, the ROS content and SOD activity in the kidney tissue were normal. In contrast, the ROS levels due to oxidative stress (Figure 5A) were significantly increased, and the antioxidative SOD activity (Figure 5B) was reduced (*p* < 0.05) in the salt-induced SHRSP rats (SHRSP+NaCl group). However, in the salt-treated SHRSP rats (SHRSP+NaCl+KPs group), the SOD activity was increased, and the ROS levels were significantly deceased after the KPs administration for 4 weeks.

### 3.7. KPs Reduced Salt-Induced Renal Inflammatory Cytokines, the NLRP3 Inflammasome and Fibrotic Activation in SHRSP Rats

**Figure 6:**
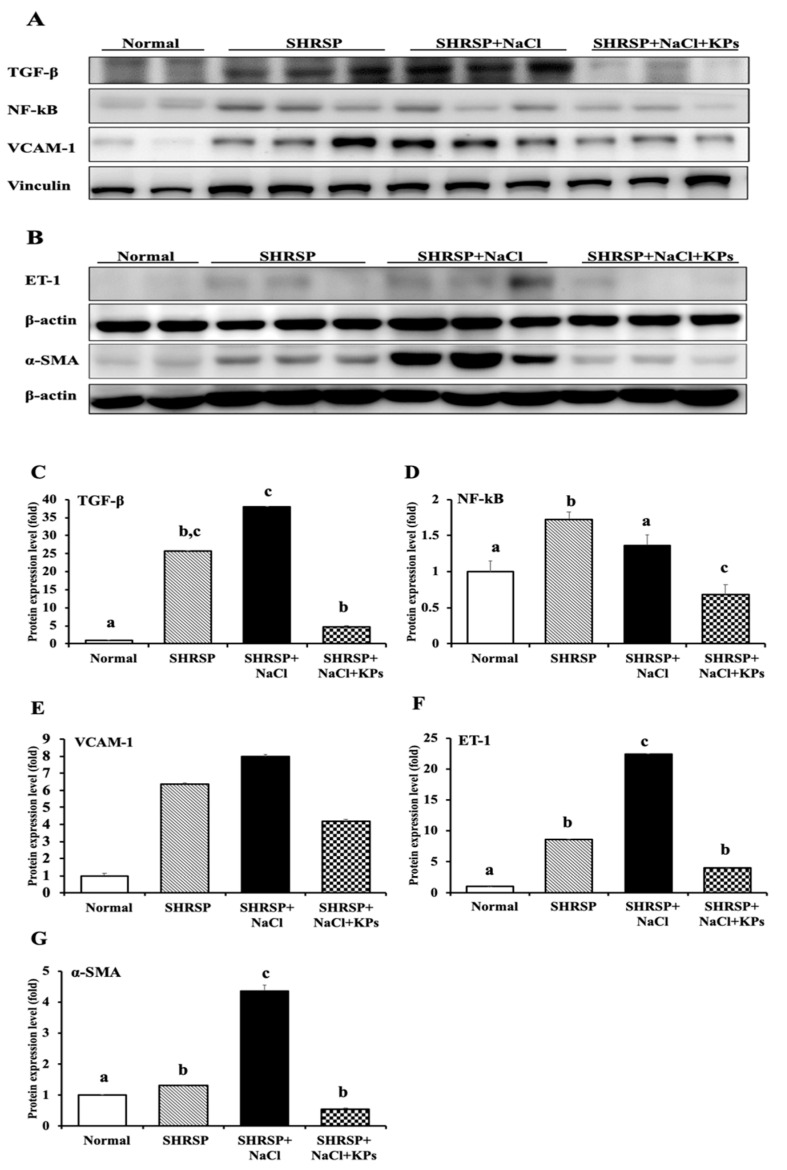
Effect of KPs on inflammation and endothelial and fibrosis biomarker expression in the kidney tissues of WKY and SHRSP rats, as determined by Western blot analysis. (**A**) The levels of inflammatory factors (TGF-β and NF-κB) and a renovascular adhesion molecule (VCAM-1) were increased in the kidney tissues of SHRSP rats with or without salt induction compared to the WKY (normal) rats but reduced in the SHRSP+NaCl+KPs group after the KPs treatment. (**B**) The levels of a fibrosis biomarker (α-SMA) and endothelial biomarker (ET-1) were increased in the kidney tissue of the SHRSP+NaCl group compared to the WKY (normal) rats and the SHRSP group. The high α-SMA and ET-1 expressions in the SHRSP+NaCl group were reversed by the KPs treatment (SHRSP+NaCl+KPs group). Quantitative analyses of the results of the Western blot analysis of TGF-β (**C**), NF-κB (**D**), VCAM-1 (**E**), ET-1 (**F**), and α-SMA (**G**) were carried out with ImageQuant^TM^ TL8.1 analysis software. Data are presented as the means ± SEMs (Normal, *n* = 5; SHRSP, *n* = 5; SHRSP+NaCl, *n* = 6; SHRSP+NaCl+KPs, *n* = 6) and were analyzed by one-way ANOVA. Data within columns that are not labeled with the same letter are significantly different (*p* < 0.05), as determined using Duncan’s test.

To elucidate the mechanisms of salt-induced renal dysfunction, inflammatory signaling due to TNF-α, TGF-β, and NF-κB protein expression in the kidneys was studied. The cytokine TNF-α in the kidney tissue lysate was significantly upregulated by 150% in the salt-treated SHRSP rats compared with the untreated SHRSP rats (*p* < 0.05), as detected with a cytometric bead array system (Supplementary Figure S2). A Western blot analysis of SHRSP rat kidney tissues also revealed that the proinflammatory cytokines TGF-β and NF-κB were significantly upregulated in the SHRSP+NaCl group compared to the Normal (WKY) group (*p* < 0.05; Figure 6A). However, the treatment with KPs significantly decreased the TGF-β (Figure 6C) and NF-κB (Figure 6D) expression compared to that in the SHRSP+NaCl group (*p* < 0.05). The renovascular damage markers VCAM-1, an adhesion protein, and endothelial ET-1 were both significantly elevated in the salt-treated SHRSP rats, whereas the oral administration of KPs for 4 weeks significantly decreased the VCAM-1 (Figure 6E) and ET-1 (Figure 6F) expression compared to that in the SHRSP+NaCl group (*p* < 0.05). The protein expression of α-SMA was examined as a marker of activated fibroblasts (Figure 6B), characteristic of some connective tissue diseases. In the SHRSP rats, a high salt intake for 4 weeks caused a significant increase in the accumulation of α-SMA compared to that of the untreated SHRSP rats (*p* < 0.05) (Figure 6G). However, the treatment with KPs significantly decreased the α-SMA expression compared to that in the SHRSP+NaCl group (*p* < 0.05).

**Figure 7:**
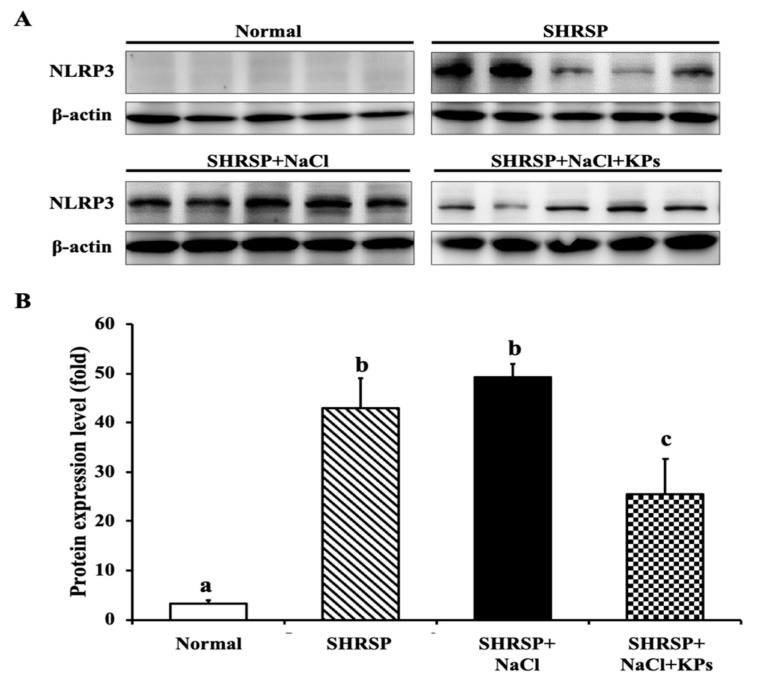
KPs reduced the NLRP3 inflammasome expression in the kidneys of salt-treated SHRSP rats, as determined by Western blot analysis. (**A**) NLRP3 inflammasome protein expression was elevated in 60% (*3/5*) of the untreated SHRSP rats and 100% (*5/5*) of the salt-treated SHRSP rats, but only a small amount of NLRP3 inflammasome was present in the SHRSP rats administered KPs. (**B**) Quantitative analysis of the results of the Western blot analysis to determine the NLRP3 protein expression level by ImageQuant^TM^ TL8.1 analysis software. Data are presented as the means ± SEMs (Normal, *n* = 5; SHRSP, *n* = 5; SHRSP+NaCl, *n* = 6; SHRSP+NaCl+KPs, *n* = 6) and were analyzed by one-way ANOVA. Data within columns that are not labeled with the same letter are significantly different (*p* < 0.05), as determined using Duncan’s test.

Furthermore, NLRP3, which senses danger signals, including bacterial lipopolysaccharides (LPS), ROS, and high concentrations of salt, and then responds by initiating an inflammatory cascade, was detected. The NLRP3 inflammasome protein level was elevated in 60% of the untreated and 100% of the salt-treated SHRSP rats (Figure 7A). The KPs administration significantly reduced the NLRP3 activation in the salt-treated SHRSP rats (*p* < 0.05; Figure 7B).

## 4. Discussion

**Figure 8:**
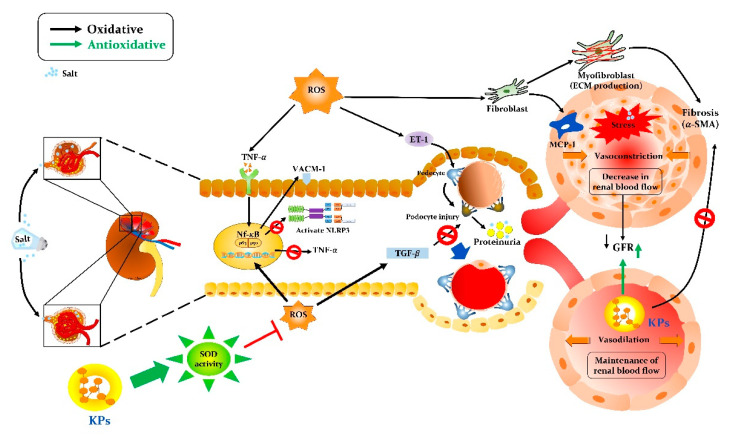
Schematic mechanism by which kefir peptides (KPs) ameliorate salt-induced renal damage and dysfunction in the SHRSP rat model. Kefir peptides may protect against renal damage and dysfunction through their antioxidant function, which increases the SOD activity and reduces ROS accumulation; anti-inflammatory function, which decreases NF-κB, TNF-α, and NRLP3 inflammatory factors; and antifibrotic function, which inhibits TGF-β, MCP-1, ET-1, and α-SMA generation, attenuating interlobular arterial disorders and maintaining a normal glomerular filtration rate and renal blood flow.

Chronic exposure to excessive salt, as occurs with a Western diet or the consumption of fast food, impairs the ability of the kidneys to maintain the pressure-natriuresis relationship, resulting in various renal injuries and hypertension [29]. The number of patients with renal damage from CKD is increasing worldwide, and CKD is also associated with an increased risk of sudden death [30]. Although the precise mechanisms by which high salt promotes renal dysfunction are controversial, there is growing evidence to suggest that inflammation plays an important role [29]. In this study, we established a salt-induced nephropathy animal model in SHRSP rats and demonstrated that the oral administration of kefir peptides could reduce renal damage and renovascular dysfunction. KPs treatment for 4 weeks protected against the progressive reduction in the renal interlobular arterial wall thickness, collagen deposition, glomerulosclerosis and tubular damage. The major new findings of this study indicate that KPs, small peptides derived from kefir fermentation, are highly effective in elevating the SOD antioxidant activity to inhibit ROS production and limiting renal inflammatory cytokines (TGF-β and NF-κB), MCP-1 (a chemoattractant), adhesive and endothelial molecules (VCAM-1 and ET-1), and fibrotic factor (α-SMA) accumulation in salt-treated SHRSP rats through the inhibition of NLRP3 and its signaling cascade. The proposed mechanisms by which KPs ameliorate salt-induced renal damage and dysfunction in SHRSP rats via anti-inflammatory, antioxidant, and antifibrotic pathways are shown in Figure 8.

Figure 1 shows that in this study, high salt-damaged kidneys were reduced in size and exhibited slight discoloration, but the abnormal kidney morphology induced by salt treatment was effectively modulated by the KPs administration. Furthermore, the treatment with KPs for 4 weeks increased body weight, suggesting that the observed improvements result from the attenuation of renal damage and dysfunction. In addition, the histopathologic examination of kidney specimens from high salt-treated SHRSP rats showed severe vascular lesions (Figure 2), tubular damage, and glomerular sclerosis, in addition to the massive accumulation of collagen (Figure 3). Our results from the high salt-treated SHRSP rats are similar to the observations of Gelosa et al. [4], who found that SHRSP rats fed a high-salt diet exhibited severe nephrosclerosis. Chao et al. [31] showed that the progression of kidney damage is characterized by phenomena such as the inadequate filtration of proteins (proteinuria), apoptosis, inflammatory cell recruitment, and the presence of ECM proteins in the interstitium, and that kidney fibrosis is the final contributing factor to renal dysfunction. However, treatment with 200 mg/kg/day KPs for 4 weeks completely prevented the development of renal lesions and abolished kidney fibrosis and collagen accumulation, which was not observed in the SHRSP rats administered high salt alone. Thus, the renal dysfunction observed in the salt-treated SHRSP rats was reversed after the KPs treatment.

In addition, high-salt drinking water increased the thickness of the interlobular arterial walls in SHRSP rats (Figure 2). Accompanying this increased thickness were notable inflammatory cell infiltration in the adventitia, an increased number of smooth muscle cell layers in the media, and a decreased diameter of the vessel lumen. These effects are similar to those of another animal model induced by the deoxycorticosterone acetate (DOCA)-salt method [32]. Treatment with DOCA-salt for 4 weeks produced progressive increases in systolic blood pressure and related organ damage, including cardiovascular hypertrophy, renal dysfunction, and renal tissue injury in rats. The histopathological examination of the kidneys of DOCA-salt rats revealed tubular, glomerular, and vascular lesions. When renal vascular hypertrophy was evaluated, significant increases in the wall thickness, wall area, and the wall-to-lumen ratio were observed in the DOCA-salt-treated rats compared with sham rats [33]. However, the interlobular arterial thickness was obviously reduced by the KPs treatment in the salt-treated SHRSP rats. ET-1, the most powerful endogenous vasoconstrictive peptide, is involved in the pathogenesis of CKD. This peptide is produced by tubular epithelial cells in response to activation by filtered proteins and involved in the development of renal scarring [34]. ET-1 also contributes to the pathogenesis and maintenance of hypertension and arterial stiffness. In this study, we found that the ET-1 expression was increased in the kidneys of SHRSP rats that received a high-salt diet, and treatment with KPs significantly decreased the ET-1 expression (Figure 4 and Figure 6). Thus, KPs ameliorated interlobular arterial disorders that cause hypertension and renal damage through inhibiting the ET-1 overexpression.

The highest urine protein-to-creatinine ratio (UPC) associated with protein-losing glomerulopathy is observed in glomerular amyloidosis. Protein-losing nephropathy is a common cause of persistent hypertension [35]. Thus, UPC is a good indicator of kidney disease, regardless of disease progression. The salt-treated SHRSP rats exhibited 400% more UPC in their urine compared with that in the untreated SHRSP rats; however, the treatment with KPs restored the UPC values (Table 1). Furthermore, CKD is defined by detection of a matrix of biomarkers, specifically, the glomerular filtration rate (GFR) and albuminuria in the context of manifestations of kidney damage [36]. A previous study showed that a normal glomerular filtration rate and renal blood flow were maintained in young SHRSP rats (<12 weeks old) in part by an autoregulatory mechanism, whereas in middle-aged (>24 weeks old) SHRSP rats the preglomerular vessels were sclerotic, reducing renal blood flow, and the glomerular permeability to macromolecules was enhanced, leading to tubulointerstitial inflammation and finally glomerulosclerosis [37]. In this study, compared to untreated SHRSP rats, the salt-induced aged SHRSP rats (50–60 weeks old) not only exhibited a significant reduction in the glomerular filtration ability by 64% and a 70% reduction in urine creatinine but also increased the urine fractional excretion of electrolytes (FeNa and FeCl). Glomerulosclerosis, tubular atrophy, and the dilatation of the Bowman’s space shown by histological examination of salt-treated SHRSP rats (Table 2) were dramatically restored by the KPs administration for 4 weeks.

Enhanced activation of TGF-β, NF-κB, and VCAM-1 signaling is a key mechanism of kidney injury. TGF-β activity not only plays an important role in inflammation and the pathophysiology of tubulointerstitial fibrosis but also affects various cell types involved in renal damage, such as tubular epithelial cells, renal fibroblasts, macrophages, and endothelial cells [38]. Macrophage accumulation in the kidney further directly increases the TGF-β activity by both producing large amounts of TGF-β and generating ROS, which activate latent TGF-β [39]. A previous study suggested that a high-salt diet in rats led to increased oxidative stress in the microcirculation and altered the ratio between ROS and the levels of antioxidative enzymes (such as SOD and catalase) [40]. Accordingly, NF-κB can regulate the expression of a large number of target genes involved in the immune and inflammatory response, apoptosis, cell proliferation, differentiation, and survival. The activated expression of the proinflammatory molecule VCAM-1 is the ultimate consequence of renovascular inflammation mediated by the NF-κB-dependent signaling pathway [41]. Additionally, MCP-1 is produced by mesangial and tubular epithelial cells, predominantly causing renal interstitial inflammation, tubular atrophy, and interstitial fibrosis [42]. In the present study, the MCP-1 expression was significantly elevated in the salt-treated SHRSP rats, and this increased expression was associated with increased macrophage infiltration. However, treatment with KPs markedly decreased salt-induced inflammation in the kidney. Thus, these results revealed that KPs attenuated the kidney MCP-1 expression, possibly leading to the recovery of glomerular and tubulointerstitial lesions, in the salt-treated SHRSP rats.

Our results showed that KPs treatment significantly reduced oxidative stress and increased the SOD activity in the kidneys of salt-induced SHRSP rats. However, Normal (WKY) and SHRSP is a similar level for ROS/RNS as an index of oxidative stress, whereas the SOD activity of SHRSP is lower than Normal (Figure 5). Other studies support our findings and reported that the total SOD activity was decreased in the normal diet-fed SHRSP rats compared with WKY rats, while the levels of oxidative stress were highly accumulated in the brain than serum or kidneys of SHRSP [43,44], this could be the plausible cause for the significant increase in the ROS/RNS level in the kidneys of SHRSP group compared with Normal group in our experimental model. Furthermore, it was reported that SHRSP exhibits salt-sensitivity, markedly increased vascular release of superoxide, and decreased total plasma and kidney antioxidative capacity [45].

The α-SMA isoform plays an important role in fibrogenesis. Renal fibroblasts are a heterogeneous population, and a subset of these fibroblasts are myofibroblasts, which are identified and defined based on SMA expression [46]. Myofibroblasts are the site of extracellular matrix (ECM) production during fibrosis in the kidney. Accordingly, interstitial SMA and myofibroblasts are significantly related [47]. Thus, SMA expression is the best prognostic indicator of glomerulonephritis disease progression [48]. Excessive inflammatory responses at the early stages of kidney injury are thought to be important factors that contribute to matrix deposition and fibrosis [49]. The infiltration of inflammatory cells, including monocytes and macrophages, and fibrosis are found in kidney tissues after injury [50]. Therefore, in this study, we identified obvious renal injury and tubulointerstitial fibrosis in the kidney tissues of salt-treated SHRSP rats (SHRSP+NaCl group). Additionally, inflammasomes are crucial mediators of inflammation in numerous chronic inflammatory diseases, including osteoarthritis, metabolic syndrome, atherosclerosis, and age-related muscular degeneration [29,51]. Recent studies using a variety of animal models have shown that the development of hypertension and associated renal inflammation is dependent on the presence of functional NLRP3 inflammasome signaling [52,53]. In this study, we found that, compared to untreated SHRSP rats, 100% of salt-treated SHRSP rats exhibited higher levels of NLRP3 in their renal tissue, but the NLRP3 levels were significantly decreased after KPs administration. Therefore, KPs may confer benefits by improving renal hemodynamics, reducing proteinuria, and reducing renovascular risk through inhibiting NLRP3 inflammasome signaling.

## 5. Conclusions

In this study, salt-induced SHRSP rats which exhibited renal damage and dysfunction were successfully established as an animal model that closely mimics clinical CKD. The results from this study demonstrated that treatment with KPs attenuated salt-induced severe renovascular lesions, tubular damage, renal fibrosis, and glomerular sclerosis. Further results revealed that KPs could suppress oxidative stress and NLRP3 and TGF-β signaling and reduce urine UPC and the fractional excretion of electrolytes through their anti-inflammatory, antioxidant, and antifibrotic abilities. Therefore, the results of this study suggest that KPs ameliorate high salt-induced renal damage and dysfunction by inhibiting NLRP3, ROS, and α-SMA, and that KPs might be a promising protective nutraceutical agent against high salt-induced renovascular-related diseases.

## Figures and Tables

**Table 1:** Effects of kefir peptides (KPs) on the body weight, organ index, BUN, CRE, CRE/BUN ratio, urine fractional excretion of electrolytes (FeX), UCRE, UPRO, and UPC in different treatment groups of SHRSP rats.

	Parameters	Normal	SHRSP	SHRSP+NaCl	SHRSP+NaCl+KPs
	Body weight (g)	398 ± 5.82 ^a^	322 ± 8.29 ^b^	202 ± 7.19 ^c^	317 ± 5.86 ^b^
	Kidneys index (%) ^1^	0.677 ± 0.019 ^a^	0.914 ± 0.048 ^bc^	0.936 ± 0.076 ^c^	0.835 ± 0.021 ^bc^
Serum	BUN (mg/dl)	15.8 ± 0.3 ^a^	26.8 ± 2.0 ^b^	31.7 ± 3.1 ^b^	30.4 ± 4.5 ^b^
	CRE (mg/dl)	0.4 ± 0.03 ^a^	0.4 ± 0.04 ^a^	0.5 ± 0.08 ^a^	0.4 ± 0.04 ^a^
	CRE/BUN ratio	0.022 ± 0.001 ^a^	0.015 ± 0.001 ^a^	0.015 ± 0.002 ^a^	0.014 ± 0.001 ^a^
	GFR/g kidney weight (ml/min/g)	2.19 ± 0.14 ^a^	1.69 ± 0.31 ^b^	0.71 ± 0.49 ^c^	1.60 ± 0.13 ^b^
Urine	FeNa (%) ^2^	0.05 ± 0.01 ^a^	0.23 ± 0.05 ^a^	1.47 ± 0.52 ^b^	0.23 ± 0.02 ^a^
	FeK (%)	7.32 ± 0.66 ^a^	8.68 ± 1.62 ^a^	5.91 ± 0.82 ^a^	8.65 ± 1.31 ^a^
	FeCl (%)	0.16 ± 0.01 ^a^	0.44 ± 0.08 ^a^	2.33 ± 0.67 ^b^	0.39 ± 0.05 ^a^
	UCRE (mg/dl)	24 ± 3.25 ^a^	63 ± 3.69 ^b^	20 ± 2.87 ^a^	55 ± 3.45 ^b^
	UPRO (mg/dl)	42 ± 2.74 ^a^	764 ± 73.59 ^b^	901 ± 66.71 ^c^	762 ± 26.0 ^b^
	UPC	0.57 ± 0.04 ^a^	12.1 ± 1.16 ^b^	49.3 ± 8.72 ^c^	14.0 ± 0.76 ^b^

^1^ Organ index (%) = [organ weight (g)/final body weight (g)] × 100. ^2^ Fe (Na, K, Cl) (%) = [(Na, K, Cl) urine to creatinine ratio/ (Na, K, Cl) plasma to creatinine ratio] × 100. BUN, blood urea nitrogen; CRE, creatinine; UCRE, urine creatinine; UPRO, urine protein; UPC, urine protein-to-creatinine ratio. Data are presented as the means ± SEMs (Normal, *n* = 5; SHRSP, *n* = 5; SHRSP+NaCl, *n* = 6; SHRSP+NaCl+KPs, *n* = 6). ^a,b,c^ Data in the same row without the same superscript letters are significantly different (*p* < 0.05), as determined using Duncan’s test.

**Table 2:** Summary of the pathological scores related to kidney disease in different treatment groups of SHRSP rats.

Lesions ^1^	Group ^2^			
	Normal	SHRSP	SHRSP+NaCl	SHRSP+NaCl+KPs
Afferent arteriolopathy, segmental glomerulosclerosis, and tubular atrophy in the juxtamedullary cortex	0 ^a^	1.3 ± 0.63 ^a^	2.5 ± 0.29 ^b^	0.7 ± 0.33 ^a^
Cast, hyaline, tubule, focal	0 ^a^	2.0 ± 0.41 ^b^	3.0 ± 0.41 ^b^	2.0 ± 0.00 ^b^
Chronic progressive nephrosis	0 ^a^	2.5 ± 0.29 ^c^	3.3 ± 0.25 ^c^	1.3 ± 0.67 ^b^
Hypertrophy, media, arterioles, focal	0 ^a^	1.8 ± 0.85 ^b^	3.0 ± 0.00 ^b^	0.3 ± 0.33 ^a^

^1^ The degree of the lesions was scored from 1 to 5 depending on severity: 1 = minimal (< 1%); 2 = slight (1–25%); 3 = moderate (26–50%); 4 = moderate/severe (51–75%); and 5 = severe/high (76–100%). ^2^ Data = means ± SEMs (Normal, *n* = 5; SHRSP, *n* = 5; SHRSP+NaCl, *n* = 6; SHRSP+NaCl+KPs, *n* = 6). ^a,b,c^ Data in the same row without the same superscript letters are significantly different (*p* < 0.05), as determined using Duncan’s test.

## Supplementary Materials

The following are available online at https://www.mdpi.com/2076-3921/9/9/790/s1; Figure S1: Different-dosage pretreatments of kefir peptides in salt-untreated SHRSP rats to observe the reduction of oxidative stress, inflammation reaction, and the increase of kidney SOD activity; Figure S2: KPs reduced activity of the inflammatory factor TNF-α in the kidney tissue of WKY and SHRSP rats, as determined by cytometric bead array. Table S1: The main compositions of kefir peptides (KPs); Table S2: Effects of KPs on UPRO, UCRE and UPC measurements weekly in different treatment groups of SHRSP rats.
