# Supplementary material for: Anti-Inflammatory, Antioxidant, and Antifibrotic Effects of Kefir Peptides on Salt-Induced Renal Vascular Damage and Dysfunction in Aged Stroke-Prone Spontaneously Hypertensive Rats
Source: Antioxidants (Basel). 2020 Aug 26;9(9):790. doi: 10.3390/antiox9090790 (PMC7555286; doi:10.3390/antiox9090790)
Supplement: Supplementary file 1 [file antioxidants-09-00790-s001.pdf]

Supplementary Table S1. The main compositions of kefir peptides (KPs)

Item	Content
Total peptides	15.48 g/100g
>30 kD KPs	9.23 g/100g
3-30 kD KPs	4.53 g/100g
<3 kD KPs	1.72 g/100g
....	....
Energy	487 kcal/100g
Protein	23.1 g/100g
Fat	26.1 g/100g
Carbohydrates	40 g/100g
Sodium	0.28 g/100g
Sugars	10.6 g/100g

Supplementary Table S2. Effects of KPs on UPRO, UCRE and UPC measurements weekly in different treatment groups of SHRSP rats

Week	Normal			SHRSP			SHRSP+NaCl			SHRSP+NaCl+KPs		
	UPRO	UCRE	UPC	UPRO	UCRE	UPC	UPRO	UCRE	UPC	UPRO	UCRE	UPC
1	44±4.01	26±2.44	0.59±0.03[a]	698±24.78	55±2.63	11.9±1.05[b]	762±53.07	55±3.87	13.9±0.89[b]	878±102.3	69±6.94	12.7±0.45[b]
2	44±3.55	27±3.82	0.61±0.06[a]	704±32.65	58±4.22	12.5±1.10[b]	655±33.64	23±0.79	29.0±2.04[c]	805±44.6	58±3.24	13.9±0.76[b]
3	43±3.40	25±4.02	0.57±0.06[a]	722±81.14	56±3.53	12.8±1.22[b]	643±50.57	18±1.71	37.8±3.17[c]	757±62.1	57±2.15	13.4±1.13[b]
4	43±2.74	25±3.25	0.57±0.04[a]	764±73.59	63±3.69	12.1±1.16[b]	901±66.71	20±2.87	49.3±8.72[c]	762±26.0	55±3.45	14.0±0.76[b]

UCRE, urine creatinine; UPRO, urine protein; UPC, urine protein-to-creatinine ratio.

Data are presented as the means ± SEMs.

[a,b,c] Data in the same row without the same superscript letters are significantly different (P<0.05), as determined using Duncan's test.

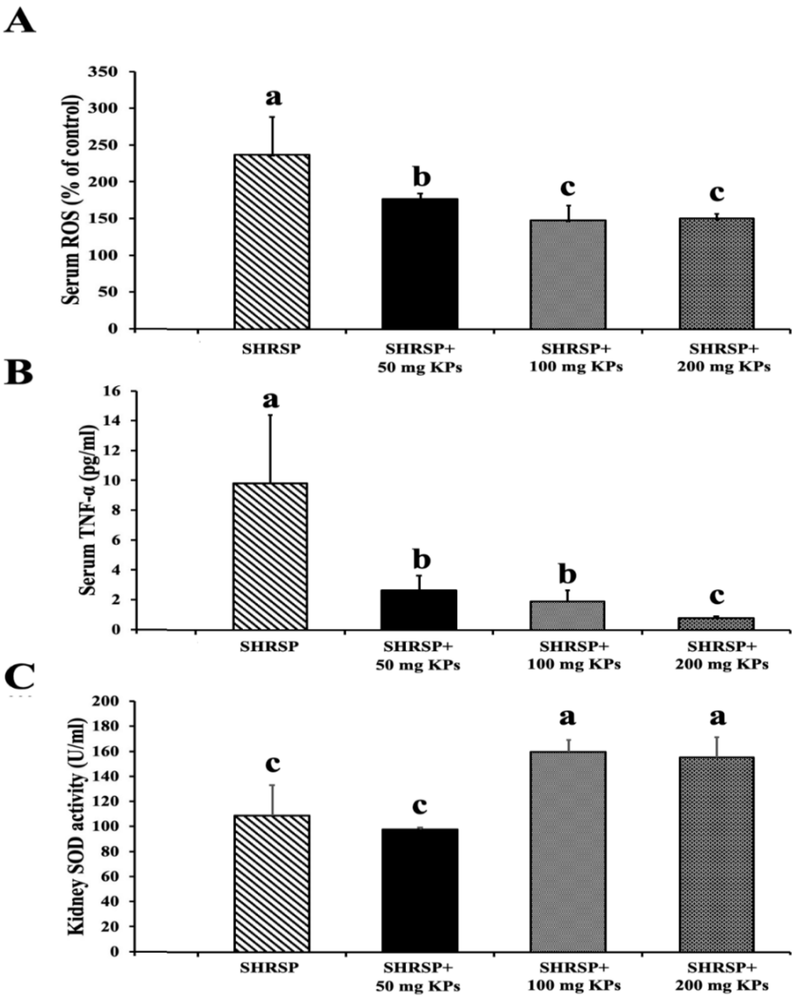

Supplementary Figure S1. Different-dosage pretreatments of kefir peptides in salt-untreated SHRSP rats to observe the reduction of oxidative stress, inflammation reaction and the increase of kidney SOD activity. Three different doses of kefir peptides (KPs), 50 mg/kg, 100 mg/kg and 200 mg/kg, were tested in salt-untreated SHRSP rats and antioxidant and anti-inflammatory effects were measured by serum ROS accumulation **(A),** serum TNF-α accumulation **(B)** and kidney SOD activity **(C).** Results showed that the treatment with 200 mg/kg KPs exhibits more effective on reduction of the ROS and TNF-α levels than that of 50 mg/kg and 100 mg/kg KPs treatments. Therefore, we selected the dose of 200 mg/kg KPs in the entire experiment. Data are presented as the means ± SEMs and were analyzed by one-way ANOVA. Data within columns that are not labeled with the same letter are significantly different (P<0.05), as determined using Duncan's test.

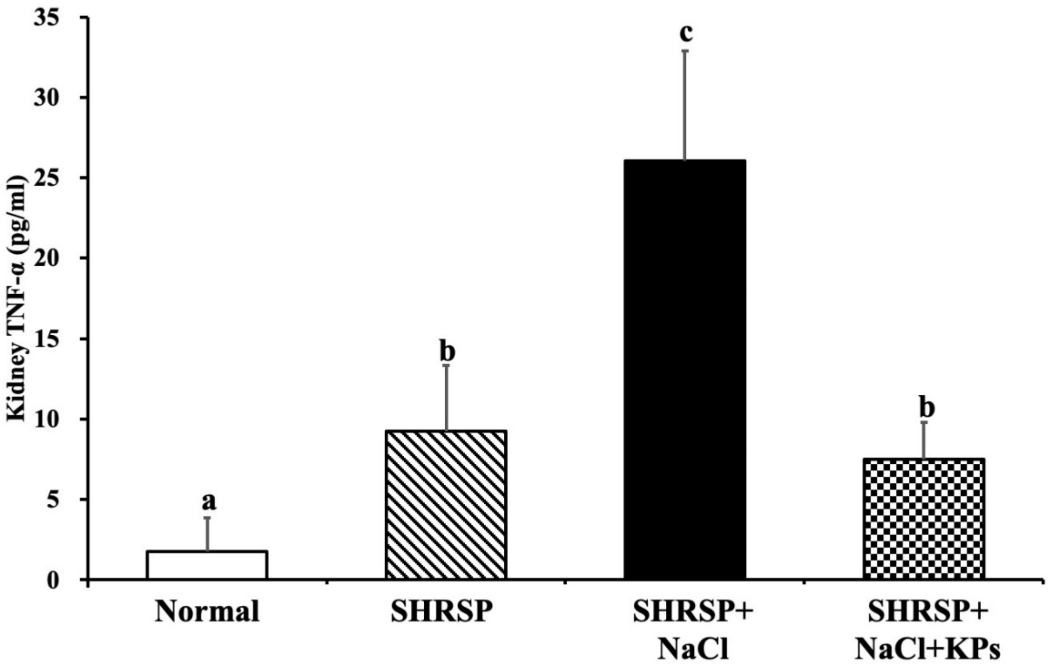

Supplementary Figure S2. KPs reduced activity of the inflammatory factor TNF-α in the kidney tissue of WKY and SHRSP rats, as determined by cytometric bead array. The supernatants of kidney tissue lysates obtained from WKY and SHRSP rats were subjected to an inflammatory TNF-α activity assay using a Cytometric Bead Array (BD Biosciences, San Jose, CA, USA). Greater TNF-α activity was observed in the SHRSP+NaCl group compared to the WKY (normal, N=5) rats and SHRSP group (N=6). The increased TNF-α activity in the SHRSP+NaCl group (N=6) could be reversed by KPs treatment (SHRSP+NaCl+KPs group). Data are presented as the means ± SEMs and were analyzed by one-way ANOVA. Data within columns that are not labeled with the same letter are significantly different ($P<0.05$), as determined using Duncan's test.
